# Impact of statins therapy on morphological changes in lipid-rich plaques stratified by 10-Year framingham risk score: A serial optical coherence tomography study

**DOI:** 10.18632/oncotarget.16172

**Published:** 2017-03-14

**Authors:** Yinchun Zhu, Lin Lin, Chao Wang, Haibo Jia, Sining Hu, Lulu Li, Xiling Zhang, Gonghui Zheng, Yan Wang, Rong Sun, Abigail Afolabi, Irina Mustafina, Jingbo Hou, Shaosong Zhang, Bo Yu

**Affiliations:** ^1^ Department of Cardiology, The Second Affiliated Hospital of Harbin Medical University, The Key Laboratory of Myocardial Ischemia, Chinese Ministry of Education, Harbin, China; ^2^ Department of Cardiology, The Second Affiliated Hospital of Harbin Medical University, Harbin, China

**Keywords:** statins therapy, morphological changes, lipid-rich plaques, framingham risk score, optical coherence tomography

## Abstract

The aim of the study was to investigate the impact of statins therapy on morphological changes of lipid-rich plaques by OCT (optical coherence tomography) in patients with known CHD (coronary heart disease), stratified by FRS. Ninety-seven lipid-rich plaques from sixty-nine patients who received statins therapy and underwent serial OCT images (baseline, 6-month and 12-month) were divided into 2 groups according to the FRS (framingham risk score): low risk group A (FRS<10%, N=35, n=45), moderate to high risk group B (FRS≥10%, N=34, n=52). Fibrous cap thickness (FCT) was measured at its thinnest part 3 times. Baseline characteristics were not different between the 2 groups. FCT sustained increased from baseline to 6-month and 12-month follow up in both group A (59.8±20.4μm, 118.3±62.5μm, and 158.8±83.4μm respectively, *P*<0.001) and group B (62.2±16.8μm, 125.1±78.7μm, 163.8±75.5μm respectively, *P*<0.001). Lipid index was significantly decreased in both group A (1862.1±1164.5, 1530.3±1108.7, 1322.9±1080.4, *P*<0.001) and group B (1646.6±958.5, 1535.1±1049.1, 1258.6±1045, *P*=0.016). The incidence of TCFA was decreased statistically in both group A (*P* <0.001) and group B (*P* <0.001). The patients with known CHD can equivalently benefit from statins therapy by stabilizing the lipid-rich plaques. Patients with moderate to high FRS might benefit more within the first year from event time.

## INTRODUCTION

Framingham risk score (FRS), as we known, is a 10-year risk of cardiovascular events which was calculated by sex, age, current smoking status, systolic blood pressure, total cholesterol and high density lipoprotein (HDL) cholesterol [1, 2]. Furthermore, FRS is more reliable to predict future cardiovascular risk [3] compared with other scoring systems, such as Prospective Cardiovascular Münster score, Systematic Coronary Risk Evaluation, Syntax score and so on [1, 4]. It is useful to make a decision in lipid-lowering therapy for coronary heart disease (CHD)[3]. Risk is low if the FRS is less than 10%, moderate if it is 10-20%, and high if it is more than 20%.

The most common lipid-lowering therapy was statins therapy which has been recognized as an effective approach to improve the clinical outcomes of CHD patients by stabilizing vulnerable plaques [5, 6]. Intravascular ultrasound (IVUS ) studies have demonstrated that statins can prevent the plaque progression and reverse plaque volume [7, 8]. Optical coherence tomography (OCT) has been validated to assess the plaque vulnerability and morphological changes due to its high resolution (10–20μm). Komukaietal and Takaradaetal have shown that accompanied with the level of low density lipoprotein (LDL) decreased under statins therapy, plaque tend to be stabilized regarding the increase of fibrous cap thickness (FCT) and the decrease of macrophage infiltration which are considered as the features of plaque vulnerability [9, 10].

Whether known CHD patients with different FRS can benefit equally from statins therapy after event is unknow. Therefore, in this study, we aimed to investigate the impact of statins on morphological changes of lipid-rich plaques by OCT in patients with CHD, stratified by FRS.

## RESULTS

### Baseline patient clinical characteristics

A total of 97 lipid rich plaques (LRPs) were detected in 69 patients. There were 45 plaques from 35 patients in group A and 52 plaques from 34 patients in group B. Baseline patient characteristics are shown in Table 1. There was no significant difference between the 2 groups except for the elder age (52.1±9.7 vs. 59.4±7.6, *P*=0.001) and higher prevalence of male (48.6% vs.76.5%, *P*=0.017), hypertension (51.4% vs.76.5%, *P*=0.030) and smoking (34.3% vs. 61.8%, *P*=0.022) in group B. In addition, all the 3 therapeutic dose is not statistically significant between the 2 groups (AT 60: AT 20: RT 10= 40.4%: 25.7%: 34.3% vs. 38.2%: 29.4%: 32.4%, *P*=0.942).

**Table 1:** Baseline patient clinical characteristics of the 2 groups

	low FRS group (N=35)	moderate to high FRS group (N=34)	P value
Age,year	52.1±9.7	59.4±7.6	0.001
Male gender	17(48.6)	26(76.5)	0.017
HTN	18(51.4)	26(76.5)	0.030
DM	20(57.1)	14(41.2)	0.185
HL	11(31.4)	10(29.4)	0.856
Smoking	12(34.3)	21(61.8)	0.022
Proir MI	7(20.0)	8(23.5)	0.722
PriorPCI	4(11.4)	8(23.5)	0.185
ACEI/ARB	15(42.9)	16(47.1)	0.726
Beta blockor	18(51.4)	24(70.6)	0.103
CCB	6(17.1)	13(38.2)	0.50
Clopidogrel	35(100)	33(97.1)	0.493
ASA	35(100)	33(97.1)	0.493

Continous variables are expressed as mean±SD; Categorical variables are expressed as number (percentage). *P*<0.05 was considered statistically significant.

HTN=hypertention; DM=diabetes mellitus; HL=hyperlipidemia; MI=myocardial infarction; PCI=percutaneous coronary intervention; ACEI=angiotensin-converting enzyme inhibitor; ARB=angiotensin II receptor blocker; CCB=calcium channel blockers; ASA= aspirin.

### Angiographic findings

**Figure 1:**
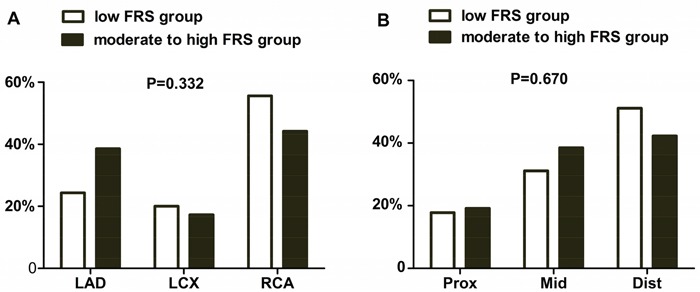
The angiographic distribution of plaques between the 2 groups Figure A showed the distribution of plaques in the 3 coronary arteries (LAD, LCX and RCA ). It was 24.4%, 20.0%, 55.6% for low risk group A ( white bar) and 38.5%, 17.3%, 44.2% for moderate to high group B (black bar). And figure B showed the distribution of plaques on the 3 lesion segments (proximal, middle and distal). It was 17.8%, 31.1%, 51.1% for low risk group A ( white bar) and 19.2%, 38.5%, 42.3% for moderate to high group B (black bar).

Baseline angiographic characteristics are showed in Table 2. The lesion distribution of the 2 groups in the 3 coronary arteries (LAD, LCX and RCA) was not significantly different (24.4% vs. 38.5%, 20.0% vs. 17.3%, 55.6% vs.44.2%) and the distribution on the 3 lesion segments (proximal, middle and distal) was also not significantly different (17.8% vs. 19.2%, 31.1% vs.38.5%, 51.1% vs. 42.3% ) (Figure 1A and Figure 1B). The minimum lumen diameter (MLD) was significantly smaller in group B (2.1±0.6 mm vs. 1.9±0.5 mm, *P*=0.010) and the reference vessel diameter (RVD) was also smaller in group B (3.0±0.6 mm vs. 2.6±0.6 mm, *P*=0.017). While the DS% and lesion length was not significantly different between the 2 groups.

**Table 2:** Angiographic findings of the 2 groups at baseline

	low FRS group (N=35)	moderate to high FRS group (N=34)	P value
Target vessel			0.332
LAD	11(24.4)	20(38.5)	
LCX	9(20.0)	9(17.3)	
RCA	25(55.6)	23(44.2)	
Location			0.670
Prox	8(17.8)	10(19.2)	
Mid	14(31.1)	20(38.5)	
Dist	23(51.1)	22(42.3)	
MLD	2.1±0.6	1.9±0.5	0.010
RVD	3.0±0.6	2.6±0.6	0.017
DS%	27.8±10.4	29.9±10.6	0.285
LL	11.4±4.8	11.2±5.3	0.844

Continous variables are expressed as mean±SD; Categorical variables are expressed as number (percentage). *P*<0.05 was considered statistically significant.

LAD= left anterior descending; LCX= Circumflex; RCA=right coronary artery; prox=proximal segment; Mid=middle segment; Dist=distal segment; MLD= minimum lumen diameter; RVD=reference vessel diameter; DS%= diameter stenosis%. LL=lesion length.

### Changes in laboratory test

**Figure 2:**
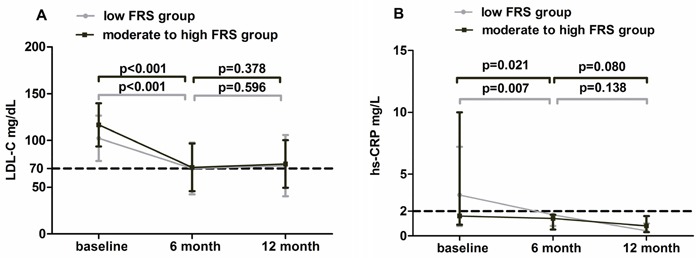
Dynamic changes in the LDL-C and hs-CRP of the 2 groups among the 3 time points **A**. LDL-C was significantly decreased in the first 6-month, and was maintained at about 70mg/dL until 1 year in both 2 groups. **B**. Serum hs-CRP was significantly reduced from baseline to 6-month (<2.0mg/L) and kept stable from 6-month to 12-month in both 2 groups.

The serum lipids, high-sensitivity C-reactive protein (hs-CRP) and glycosylated hemoglobin (Ha1C) at the 3 time points of the 2 groups in the study are shown in Table 3. Low-density lipoprotein cholesterol (LDL-C) was significantly decreased from baseline to 6-month and kept stable until 12-month in group A (102.3±24.3 mg/dL,70.0±27.6 mg/dL, 73.0±32.8 mg/dL, *P*<0.001) and group B (116.6±23.1 mg/dL, 71.2±25.5 mg/dL,74.8±25.3 mg/dL, *P*<0.001). Detailedly, there was a significantly decreased from baseline to 6-month in both 2 groups (*P*<0.001, *P*<0.001, respectively), but from 6-month to 12-month, there was no statistical significance (Figure 2A). Similar change of serum total cholesterol (TC) was also observed in group A (191.9±47.9 mg/dL, 141.3 ±34.6 mg/dL,147.9±45.5 mg/dL, *P*<0.001) and group B (205.5±34.6 mg/dL, 140.6±37.6 mg/dL,144.2±34.2 mg/dL, *P*<0.001). In addition, hs-CRP also decreased significantly from baseline to 6 month until 12 month in group A (3.3 mg/dL, 1.7 mg/dL, 0.4 mg/dL, *P*=0.002) and group B (1.6 mg/dL, 1.4 mg/dL, 0.8 mg/dL, *P*=0.003) (Figure 2B). While, there was no significant difference in serum lipids and hs-CRP at each time point between the 2 groups and the changes of laboratory data were also similar between Group A and Group B (Table 4).

**Table 3:** Laboratory test of the 2 groups at the 3 time points

	low FRS group (N=35)				moderate to high FRS group (N=34)			
	0	6	12	P	0	6	12	P
Hscrp mg/dL	3.3 (0.8, 7.2)*	1.7 (0.8,1.7)	0.4 (1.0,1.6)^&^	0.002	1.6 (0.9,10.0)*	1.4(0.5,1.7)	0.8 (0.3,1.6)^&^	0.003
Ha1C(%)	6.9±1.6	6.8±1.5	6.6±1.4	0.313	7.1±1.6	6.8±1.1	6.6±1.3	0.073
TG mg/dL	176.1 (122.1,272.6)*	131.0 (92.9,172.6)	123.0 (91.2,169.9)^&^	0.002	185.9 (137.0,220.8)*	125.2 (96.0,174.8)	130.5 (104.0,164.4)^&^	0.001
TC mg/dL	191.9±47.9 *	141.3±34.6	147.9±45.5^&^	<0.001	205.5±34.6 *	140.6±37.6	144.2±34.2	<0.001
LDL-C mg/dL	102.3±24.3 *	70.0±27.6	73.0±32.8^&^	<0.001	116.6±23.1 *	71.2±25.5	74.8±25.3	<0.001
HDL-C mg/dL	50.5±14.2	48.3±12.0	46.6±14.0	0.317	48.1±9.9	46.6±14.8	46.9±14.0	0.878

Continous variables are expressed as mean±SD; p<0.05 was considered statistically significant. *:means the difference between baseline and 6 month; &: means the difference between baseline and 12 month.

Hs-crp= high-sensitivity C-reactive protein; Ha1C = Glycosylated Hemoglobin A1c; TG= triglyceride; TC= total cholesterol; LDL-C=low-density lipoprotein-cholesterol; HDL-C=high-density lipoprotein-cholesterol.

**Table 4:** The change of laboratory test between the 2 groups among the 3 time points

	low FRS group (N=35)	moderate to high FRS group (N=34)	P value
hscrp0-6	−1.9(−6.2, 0.4)	−0.7(−10.4, 0.2)	0.990
hscrp 6-12	−0.1(−0.6, 0.1)	0(−0.7, 0.2)	0.829
hscrp 0-12	−1.8(−5.8, -0.2)	−1.1(−10.4,-0.1)	0.769
Ha1C 0-6	0±1.2	−0.3±1.4	0.333
Ha1C 6-12	−0.2±1.0	−0.2±1.1	0.885
Ha1C 0-12	−0.3±1.2	−0.6±1.6	0.434
TG 0-6	−54.9(−76.1, -8.9)	−53.5(−112.4, -13.3)	0.349
TG 6-12	−3.5(−18.6, 15.1)	−0.4(−30.5, 29.0)	0.862
TG 0-12	−54.0(−90.3, -22.1)	−62.4(−113.3,-8.9)	0.446
TC 0-6	−50.1±35.9	−65.4±43.6	0.116
TC 6-12	6.6±37.0	3.6±30.8	0.716
TC 0-12	−43.5±40.0	−61.8±40.3	0.063
LDL-C 0-6	−35.4±30.5	−42.3±34.0	0.379
LDL-C 6-12	3.0±33.0	3.6±24.0	0.928
LDL-C 0-12	−32.4±31.6	−38.7±31.3	0.413
HDL-C 0-6	1.2(−6.6, 8.2)	−3.7(−12.6, 4.1)	0.100
HDL-C 6-12	−1.5(−6.6, 3.5)	1.0(−6.4.3.7)	0.746
HDL-C 0-12	1.5(−7.3, 5.8)	−6.8(−15.4, 3.9)	0.099

Continous variables are expressed as mean±SD; p<0.05 was considered statistically significant. Hs-crp= high-sensitivity C-reactive protein; Ha1C = Glycosylated Hemoglobin A1c; TG= triglyceride; TC= total cholesterol; LDL-C=low-density lipoprotein-cholesterol; HDL-C=high-density lipoprotein-cholesterol.

### Changes of OCT parameters

**Figure 3:**
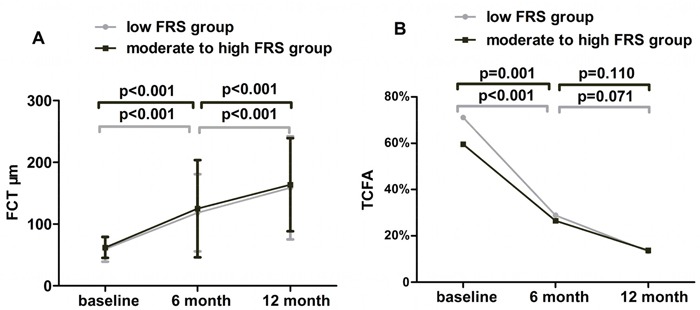
Dynamic changes in the FCT and the incidence of TCFA of the 2 groups among the 3 time points **A**. Fibrous-cap thickness was sustained increased from baseline to 12-month in both 2 groups. **B**. The incidence of TCFA was sustained decreased in both 2 groups under the lipid lowering therapy.

A total of 97 LRPs (45 in group A and 52 in group B) were included in this study. The OCT characteristics of the LRPs were showed in Table 5. FCT continuously increased from baseline to 6 month and to 12 month in group A (59.8±20.4μm, 118.3±62.5μm,158.8±83.4μm, *P*<0.001) and group B (62.2±16.8μm, 125.1±78.7μm, 163.8±75.5μm, *P*<0.001). In detail, FCT was significantly sustained growth in both 2 groups from baseline to 6-month (*P*<0.001, *P*<0.001, respectively) and from 6-month to 12-month (*P*<0.001, *P*<0.001, respectively) (Figure 3A). While there was no statistical significance in the changes of FCT at each time point between the 2 groups (Table 6). And there was also no significant difference between the two groups at baseline (*P* =0.528), 6month (*P* =0.642) and 12 month follow up (*P* =0.761). In addition, the incidence of thin-cap fibro-atheroma (TCFA) was significantly decreased in both 2 groups under the statin therapy (both *P*<0.001). For group A, the incidence of TCFA was significantly decreased from baseline to 6-month (71.1% to 28.9%, *P*<0.001) but was not different from 6-month to 12-month (28.9% to 13.3%, *P*=0.106) (Figure 3B). And it was same to group B from baseline to 6-month (59.6% to 26.5%, *P*=0.001) and from 6-month to 12-month (26.5% to 13.7%, *P*=0.110). When comparing the incidence of TCFA at the 3 time points, it was similar between group A and group B at baseline (*P* =0.237), 6-month (*P* =0.798) and 12-month (*P* =0.955). The incidence of macrophage accumulation and cholesterol crystals decreased significantly from baseline to 12 month in group B (80.8%, 61.5%, 54.9%, *P*=0.016) but not in group A (30.8%, 19.2%, 14.0%, *P*=0.036), while the change of micro-channel did not reach the statistical significance in both two groups. When comparing the incidence of macrophage accumulation, cholesterol crystals and micro-channel, it was not different between group A and group B at each time point.

**Table 5:** OCT parameters of the 2 groups at the 3 time points

	low FRS group (N=35)				moderate to high FRS group (N=34)			
	0	6	12	P	0	6	12	P
FCT	59.8±20.4*	118.3±62.5^#^	158.8±83.4^&^	<0.001	62.2±16.8*	125.1±78.7^#^	163.8±75.5^&^	<0.001
Lipid index	1862.1± 1164.5*	1530.3± 1108.7	1322.9± 1080.4^&^	<0.001	1646.6± 958.5	1535.1± 1049.1^#^	1258.6± 1045.^&^	0.016
TCFA	32(71.1)*	13(28.9)	6(13.3)^&^	<0.001	31(59.6)*	13(26.5)	7(13.7)^&^	<0.001
Macrophage	29(64.4)	30(66.7)	28(65.1)	0.975	42(80.8)*	32(61.5)	28(54.9)^&^	0.016
MC	18(40.0)	16(35.6)	10(26.3)	0.416	26(50.0)	17(34.7)	15(31.9)	0.135
Cholesteral crystal	7(15.6)	5(11.1)	3(7.1)	0.327	16(30.8)	10(19.2)	6(14.0)^&^	0.036

Continous variables are expressed as mean±SD; Categorical variables are expressed as number (percentage). p<0.05 was considered statistically significant. *:means the difference between baseline and 6 month; #: means the difference between 6 month and 12 month. &: means the difference between baseline and 12 month.

FCT= fiberous cap thickness; TCFA= Thin-cap Fibroatheroma; MC= micro-channel.

**Table 6:** The change of OCT parameters between the 2 groups among the 3 time points

	low FRS group (N=35)	moderate to high FRS group (N=34)	P value
FCT 0-6	58.6±57.9	64.0±76.4	0.709
FCT 6- 12	39.5±56.2	34.8±52.7	0.680
FCT 0-12	99.7±76.7	101.6±73.1	0.907
Lipid index 0-6	−13.0±398.1	−192.4±490.3	0.070
Lipid index 6-12	−138.8±495.7	−151.2±501.0	0.904
Lipid index 0-12	−104.1(−408.3,57.0)	−390.5(−782.0,-71.4)	0.017 e

Continous variables are expressed as mean±SD; Categorical variables are expressed as number (percentage). p<0.05 was considered statistically significant.

FCT= fiberous cap thickness.

## DISCUSSION

The major findings of the present study were as follows: First, statins therapy could decrease serum levels of atherogenic lipoproteins (LDL-C), TC, TG and inflammation biomarkers (hs-CRP ) from baseline to 6month and 12 month in the 2 groups of CHD patients regardless of risk stratification. Second, statins therapy was associated with a sustained increase in FCT regardless of risk stratification at 6-month and 12-month follow-up and the incidence of TCFA significantly decreased in both 2 groups over time. In addition, lipid index was significantly decreased in both 2 groups. Third, statins therapy could decrease macrophage content, intra-plaque micro-channel and cholesterol crystals. However, changes in macrophage content and cholesterol crystals were only observed in moderate to high risk group patients. The change of intra-plaque micro-channel did not reach the statistical significance in both 2 groups over time.

FRS was a 10-year risk score for development of cardiovascular disease and has been validated in Canada [11]. In this study, we observed that patients with moderate to high risk FRS were more elderly and had a higher rate of male, hypertension and smoking compared with group A. And it was coincident with the algorithm of FRS [1, 2]. The MLD and RVD was also significantly smaller in group B. It was caused by the diffused lesion (visual estimation) in the moderate to high FRS group patients. Although it seems that these parameters showed less atherogenic characteristics in this group, it did not reach the statistical significance. Due to the development of intravascular imaging modality, especially the superiority of OCT in diagnosing plaque vulnerability, in the current study, smaller minimal lumen diameter of angiographic findings can not be simply considered as advanced lesion. According to prospective natural-history study [12], non-culprit plaque was reported to cause future major adverse cardiovascular events even after the successful percutaneous coronary intervention (PCI), which demonstrated as TCFA, minimum lumen area and diameter stenosis. Therefore, it is crucial to better understand the morphological changes of non-culprit plaques with statins therapy. Previous studies demonstrated that secondary prevention of cardiovascular events with statins was effective [13]. In the real world, few data has reported the benefits of lipid lowering therapy on patients with different risk levels according to FRS after successful PCI. In the present study, non-culprit LRP were divided into two groups according to FRS. Our results revealed that even for lowest risk patients, statins therapy was necessary to stabilize plaques. It could stable the non-culprit plaques in CHD patients. For the moderate to high risk patients, the benefits were more significant, which suggested that statins should be routinely used after PCI regardless of the risk stratification. The present result was consistent with previous studies that statins therapy could reduce serum levels of LDL-C and hs-CRP and achieve target levels of LDL-C (LDL-C≈70mg/L) and hs-CRP (hs-CRP<2mg/L) in the first 6 months and keep stable until 12-month [9]. While the level of serum HDL and Ha1C were not correlated with FRS over the time in both 2 groups. In our previous study, the changes of OCT plaque characteristics were compared according to intensive and moderate statins therapy using OCT and IVUS [14]. The results showed that FCT increased and the prevalence of TCFA and macrophage decreased in both intensive and moderate statins therapy group. Compared with moderate lipid-lowering therapy, intensive statins therapy provided greater clinical benefit. Three different statin regimens were used in the present study and it was equally distributed in the two groups. There was no significant difference in group A between intensive statin therapy (AT60) and moderate statin therapy (AT20)(AT60: AT20= 40.4%: 25.7%) and also in group B, statin therapy was equally distributed (AT60: AT20=38.2%: 29.4%)(*P* =0.191). And in low risk group, there were no impact on the change in FCT from baseline to 12-month follow up (100.6±67.8μm vs. 89.0±85.1μm, *P* =0.700). However, in moderate to high risk group, there was significant difference in the change of FCT from baseline to 12 month follow up (143.5±81.6μm vs. 52.3±51.5μm, *P* <0.001). LRPs in moderate to high risk patients seems more sensitive to intensive statin therapy. However, the change of FCT were comparable between the two groups from baseline to 6 month and 12 month follow up (*P* =0.868, *P* =0.985, respectively) overall.

The vulnerable plaques has been demonstrated in the pathogenesis of acute coronary syndrome [15]. Plaques contributed to acute coronary events are characterized by a large plaque burden and TCFA with macrophages infiltration into the cap [15, 16]. OCT has been recognized as a high resolution imaging technique and it can recognize plaque microstructures and evaluate FCT [16, 17]. Keeping the low levels of LDL-C and hs-CRP after statins therapy can stabilize coronary plaques by increasing FCT and decreasing lipid index. It was demonstrated by the EASY-FIT study [9], which was a prospective and randomized OCT study. And in the present study, the similar findings were observed in group A and group B. FCT was significantly increased in both two groups regardless of risk stratification and lipid index were decreased in both two groups under the statins therapy with the decreasing of serum levels of atherogenic lipoproteins (LDL-C), TC, TG and inflammation biomarkers (hs-CRP) from baseline to 6month and 12 month. Interestingly, FCT was thicker and lipid index was smaller in moderate to high risk group B at baseline and follow up in the present study. But it did not reach the statistical significance. In addition, FCT changed most in the first six month and lipid index changed most in the second six month. Maybe this was limited by the study sample size and the plaques only contained LRPs. TCFA had been demonstrated as one of the most important morphological features of vulnerable plaques. TCFA had been extensively studied in both pathological and clinical study and it was an important potential predictor of plaque progression and acute coronary events. Previous OCT studies had been demonstrated that the FCT of TCFA could be thickened under the statins therapy and TCFA could be a potential target in anti-atherosclerotic therapy [10, 18]. Result of the present study was consistent with previous studies. The incidence of TCFA significantly decreased in both 2 groups regardless of risk stratification over time under the statins therapy. Previous OCT studies revealed that OCT-derived macrophages accumulation [19], micro-channel, cholesterol crystals [20, 21] were associated with plaque vulnerability. Inflammation was associated with atherogenesis and cardiovascular events. Previous studies had been demonstrated that macrophage infiltration was associated cardiovascular events and the severity of symptom [22]. In the present study, the incidence of macrophage significantly decreased in the moderate to high risk groups over time under the statins therapy. And it did not reach the statistical significance in the lowest risk group. In addition, cholesterol crystal was recognized as a feature of vulnerable plaque and was frequently associated with the major coronary acute events [21]. In the present study, the incidence of cholesterol crystal also significantly decreased in the moderate to high risk groups over time under the statins therapy. And it did not reach the statistical significance in the lowest risk group. It semms that statins therapy was more effective in stabilizing vulnerable plaques for the moderate to high risk group patients. Previous studies revealed that micro-channels were the potential predictors of atherosclerotic plaque progression and it was significantly associated with the plaque stability [18, 23]. Plaques with micro-channel had more vulnerable features in unstable angor pectoris [24] and micro-channel might be a potential therapeutic target for cardiovascular disease [25]. However, the incidence of micro-channel was not significantly decreased in the both 2 groups over time under the statins therapy. It was probably limited by the study sample size. Considering all of the CHD patients, the present study revealed that the moderate to high FRS risk stratification patients benefit most from statins therapy and it was consistent with previous studies [26–28].

Statins shoud be routinely used regardless of the risk stratification. CHD patients could equivalently benefit from statins therapy by stabilizing of the LRPs regardless of risk stratifications, stratified by 10-year FRS. Moderate to high risk patients would benefit more within the first year after event.

There were several limitations in the present study: First, this was a post-hoc sub-analysis of a randomized study, and the study sample is relatively small. Second, only LRPs were enrolled in the present study and potential selection bias is unavoidable. Third, very high-risk patients were excluded from the study, such as those with severe hepatic dysfunction or congestive heart failure. Fourth, due to the practical difficulties of imaging, very distal and ostial segments were not included in our study. Fifth, according to OCT definitions, TCFA was defined as a LRPs with FCT ≤65μm. Macrophages accumulation was defined as a region with signal-rich, distinct or confluent punctuate heterogeneous backward shadows. Due to the limitation of current OCT imaging system, we have to admitted that some fibrous plaques might have been misdiagnosed as TCFA at the presence of macrophage accumulation on the surface of fibrous atherosclerotic plaque. Lastly, the direct relationship between increase in FCT and reduction in coronary events risk remains unknown. Further studies with larger population and longterm follow-up are needed to study the impact of statins on morphological changes of LRPs, stratified by FRS.

## MATERIALS AND METHODS

### Study population

The present OCT study was a post-hoc sub-analysis of a prospective, open-labeled, randomized trial, which was performed to evaluate the progression of lipid-rich plaques treated by statins at baseline, 6-month, and 12-month follow-up [14]. A total of 120 consecutive patients who underwent OCT images were enrolled in the study between September 2009 and March 2013 at the 2nd Affiliated Hospital of Harbin Medical University. The inclusion criteria were as follows: (1) de novo lesion with luminal diameter stenosis between 20% and 70% (visual estimation) on coronary angiogram, (2) LRPs defined by OCT (FCT 120 μm and lipid arc >100), and (3) LDL-C range between 70 mg/dl and 160 mg/dl. The exclusion criteria included: (1) life expectancy <12 months, (2) contraindication to atorvastatin and rosuvastatin, (3) creatinine level >2.0 mg/dl or end-stage renal disease, (4) severe hepatic dysfunction (AST and/or ALT >3 times the upper limit of normal), and (5) congestive heart failure or left ventricle ejection fraction 35%. All the patients were treated by contemporary lipid lowering therapy: atorvastatin 60 mg/d (AT60), or atorvastatin 20 mg/d (AT20), or rosuvastatin 10 mg/d (RT10) randomly. It is worth noting that atorvastatin 60 mg/d (AT60) was thought to be the intensive lipid-lowering therapy and atorvastatin 20 mg/d (AT20) or rosuvastatin 10 mg/d (RT10) to be the moderate therapy for Asians due to the lighter body weight than Caucasians. The effective statins dose for lipid lowering therapy is thought to be lower in Asians. No patients were treated by statins or other lipid-lowering therapies before this trial. All the 6-month and 12-month follow-up OCT examinations were performed in the same segments as they were predefined at baseline and all the procedures were made by same groups in our hospital. Finally, ninety-seven lipid-rich plaques from sixty-nine patients who underwent serial OCT images (baseline, 6-month and 12-month) were divided into 2 groups according to the FRS: low risk group A: FRS<10% and moderate to high risk group B.

All the patients were provided with written informed consent and this trial was approved by the Ethics Committee of the 2^nd^ Affiliated Hospital of Harbin Medical University (Harbin, China).

### Angiography acquisition and analysis

Coronary angiography was performed by experienced operators from our hospital. All the procedures are radial approach after intracoronary injection of nitroglycerin (100-200 mg). Any non-culprit lesion with 30%-70% diameter stenosis was enrolled in the analysis. Quantitative coronary angiogram analysis (QCA) (Quantcor QCA 5.0, Pie Medical Imaging BV. Maastricht, The Netherlands) software package was used by two angiographers who were blinded to clinical information from an independent core laboratory for coronary angiograms.

### OCT image acquisition and analysis

In the present study, all the OCT procedures were performed in M2/M3 system (Saint Jude Medical, Westford, MA, USA). A 0.016-inch OCT catheter (Image Wire; LightLab Imaging/Saint Jude Medical, Westford, MA, USA) was advanced to the distal site of the target lesion through a 3-F occlusion balloon catheter. An occlusion balloon was inflated at 0.4 to 0.6 atm at the proximal site of the plaque was needed to remove red blood cells from the field of view. Lactated Ringer's solution was infused into the coronary artery from the distal tip of the occlusion balloon catheter at 0.5–2.0 mL/s by a high-pressure injector. The vessel was imaged with an automatic pullback device at 3.0 mm/s.

An offline software (Light Lab Imaging) was used for OCT image analysis at an independent core laboratory of Massachusetts General Hospital (MGH). OCT images were analyzed at 1 mm interval. All the baseline and follow-up OCT images were analyzed by two independent reviewers who were blinded to clinical information. OCT image was identified according to the criteria of the Clinical Expert Consensus Document on OCT. A third professional investigator intervened when there was any discordance between the observers and a consensus was obtained.

The target lesions were determined according to coronary angiography. At 6-month and 12-month follow-up, the corresponding segment for OCT analyzing to baseline imaging were identified on the basis of reliable anatomic marks such as the side branches, calcifications, and stent edges. All the enrolled plaques should be at least 5mm away from the stent edge.

**Figure 4:**
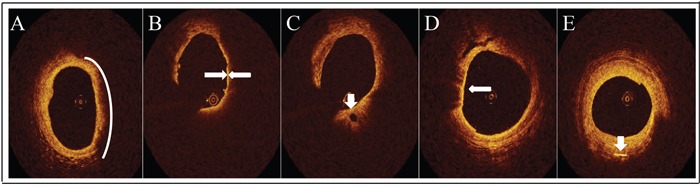
Representative OCT images **A**. Lipid core (white arc) was defined as a diffusely bordered, signal-poor region. **B**. TCFA (white arrow) was defined as a lipid-rich plaque with fibrous cap thickness≤65μm. **C**. Micro-channel (white arrow) was defined as a black hole within a plaque with a diameter of 50-300μm and can be observed on at least 3 consecutive frames. **D**. Macrophages accumulation (white arrow) was a region with signal-rich, distinct or confluent punctuate heterogeneous backward shadows. **E**. Cholesterol crystal (white arrow) was defined as linear and highly backscattering structures within the lipid-rich plaques.

Only LRPs was analyzed in this trial. On the OCT image, LRPs was semi-quantified according to the maximal lipid arc. Lipid arc was measured at every 1 mm interval throughout the entire length of each lesion and the values were averaged. All the OCT images were analyzed using the previously validated criteria for plaque characterization. LRPs was defined as the plaque with lipid content > 100° and FCT< 120 μm on OCT image. FCT covering lipid core was measured at its thinnest part 3 times and the average value of the three measurements was used for subsequent analysis. At follow up, FCT was measured at the same site according to the landmark as it was measured at baseline using the same methodology. Lipid length was also measured on longitudinal view. Lipid index (LI) was defined as the averaged lipid arc multiplied by lipid length. The characteristic of lipid core was a diffusely bordered, signal-poor region (Figure 4A). TCFA was defined as a lipid-rich plaque with FCT ≤ 65μm (Figure 4B). Microchannel was defined as a black hole within a plaque with a diameter of 50-300μm and can be observed on at least 3 consecutive frames (Figure 4C). The characteristic of macrophages accumulation was a region with signal-rich, distinct or confluent punctuate heterogeneous backward shadows (Figure 4D). Cholesterol crystals were defined as linear and highly backscattering structures within the lipid-rich plaques (Figure 4E).

### Statistical analysis

SPSS 19.0 (SPSS, IBM, Armonk, NY, USA) was used for data analysis. Continuous variables were presented as mean ± standard deviation (SD) for normally distributed variables or median (25th-75th percentiles) for non-normally distributed variables. For the normality assessment of continuous variables, the Kolmogorov–Smirnov test was used. Categorical variables were expressed as absolute numbers and percentages. For the association of qualitative variables, chi-square test was used. While for the comparison of continuous results in 2 groups over the three time points, the repeated measures analysis of variance (RM-ANOVA) with the Bonferroni correction for post-hoc comparisons was applied. Take into account that per patient may have multiple plaques, the Generalized Estimating Equations (GEE) was used when comparing the plaque characteristics in 2 groups. A *P*-value<0.05 was considered statistically significant.
